# Glymphatic system impairment in type II diabetes mellitus adults

**DOI:** 10.1038/s41598-026-36573-4

**Published:** 2026-02-04

**Authors:** Bhaswati Roy, Veronica Lubera, Kamal R. Singh, Anshita Singh, Dineth R. Karunamuni, Diana E. Kosoyan, Megan Carrier, Sarah E. Choi, Matthew J. Freeby, Rajesh Kumar

**Affiliations:** 1 Departments of Anesthesiology, University of California Los Angeles, Los Angeles, USA; 2 UCLA School of Nursing, University of California Los Angeles, Los Angeles, USA; 3 Departments of Medicine, University of California Los Angeles, Los Angeles, USA; 4 Departments of Radiological Sciences, University of California Los Angeles, Los Angeles, USA; 5 Departments of Bioengineering, University of California Los Angeles, Los Angeles, USA; 6 Brain Research Institute, University of California Los Angeles, Los Angeles, USA; 7 Department of Anesthesiology, David Geffen School of Medicine at UCLA, University of California at Los Angeles, 56-141 CHS, 10833 Le Conte Ave, Los Angeles, CA 90095-1763 USA

**Keywords:** Sleep, Cognition, ALPS index, Epworth sleepiness scale, Magnetic resonance imaging, Diffusion tensor imaging, Neurological disorders, Neuroscience

## Abstract

Type 2 diabetes mellitus (T2DM) is associated with multiple systemic complications, including cognitive decline and increased risk of neurodegenerative diseases. The glymphatic system, a brain waste-clearance pathway that can be impaired by sleep disturbances common in T2DM, has not yet been examined in the condition. Therefore, the aim was to evaluate glymphatic system in T2DM subjects using diffusion tensor imaging along the perivascular space (DTI-ALPS) index. A total of 78 T2DM adults and 106 healthy controls underwent brain MRI. Sleep issues were assessed using the Pittsburgh Sleep Quality Index (PSQI) and Epworth Sleepiness Scale (ESS), and cognition with the Montreal Cognitive Assessment (MoCA). Group differences in DTI-ALPS, sleep metrics, and MoCA scores were assessed with analysis of covariance (covariates, age, sex, and BMI). Other covariates (MoCA, ESS, and sleep apnea status) were also included to examine DTI-ALPS differences between groups, in addition to age, sex and BMI. Correlations analyses were performed to assess associations between sleep measures (PSQI and ESS), disease duration, HbA1c levels, and DTI-ALPS indices in T2DM adults. T2DM patients exhibited higher PSQI (*p* = 0.03) and ESS (*p* = 0.004), reflecting poorer sleep quality and increased daytime sleepiness. MoCA scores were significantly lower in T2DM adults (*p* = 0.001), with impairments emerged in visuospatial skills, attention, and language. Also, significantly reduced DTI-ALPS values appeared in T2DM over controls (*p* = 0.017), but no significant associations were found between DTI-ALPS index and other measures in T2DM adults. T2DM adults show impaired glymphatic function along with poor sleep quality and daytime sleepiness. The findings indicate that glymphatic dysfunction, potentially-driven by metabolic, vascular, and sleep-related disturbances may exacerbate cognitive deficits in T2DM adults.

**Supplementary Information:**

The online version contains supplementary material available at 10.1038/s41598-026-36573-4.

## Introduction

Diabetes is a widespread chronic disorder, with a global prevalence of 10.5%, affecting an estimated 537 million adults (20–79 years), and expected to rise to 783 million by 2045^1^. Type 2 diabetes mellitus (T2DM) accounts for more than 90% of total diabetes cases^2^, and is associated with significant systemic complications, including cardiovascular, renal, and brain microstructural impairments. Along with brain tissue changes^3–5^, T2DM increases the risk of cognitive decline and neurodegenerative diseases, such as Alzheimer’s disease (AD)^6^. Multiple factors, including neuroinflammation, oxidative stress, and vascular changes associated with T2DM are recognized to contribute brain tissue injury, yet the underlying mechanisms linking T2DM to progressive brain tissue changes are not fully understood. Recent studies suggest bidirectional links between T2DM and sleep disturbances, which play a critical role in cognitive health^7–9^. The glymphatic system, a recently identified waste clearance pathway in the brain, is optimally regulated during sleep and may play a critical role in maintaining brain homeostasis. While multiple mechanisms, including vascular, inflammatory, and metabolic factors have been implicated in the brain tissue changes associated with T2DM, emerging evidence suggests that glymphatic system dysfunction may represent an additional pathway linking T2DM to brain tissue microstructural alterations^10^.

The glymphatic system, which is primarily active during sleep, facilitates the removal of neurotoxic waste products, including β-amyloid and tau protein that are precursors of AD and are implicated in neurodegenerative diseases^11^. The system relies on the coordinated function of aquaporin-4 (AQP4) water channels, which are situated in astrocytic end-feet near cerebral blood vessels, to facilitate the convective flow of cerebrospinal fluid and clear interstitial solutes from brain parenchyma^12^. Multiple animal studies have shown that glymphatic clearance can be compromised by factors, such as aging^13^, high blood pressure^14^, and metabolic dysfunction^15^. However, glymphatic system function in humans with T2DM remains unexamined. Understanding the glymphatic system status in individuals with T2DM could provide insights into the mechanisms of diabetes-associated cognitive decline and the higher risks for AD, as well as suggest potential therapeutic targets.

Individuals with T2DM commonly experience various sleep issues, including poor sleep quality, insomnia, and increased daytime sleepiness, which can be worsened due to T2DM-related factors, such as obesity, inflammation, and insulin resistance. In addition, T2DM-related sleep disruptions may contribute to poor glycemic control, thereby creating a bidirectional relationships^7^. Sleep impairment has been associated with the onset of cognitive decline at an earlier age, dementia, and increased risk of developing AD^16,17^. More specifically, a recent meta-analysis conducted in T2DM patients with and without sleep issues showed 1.55-, 1.65-, and 3.78-fold increased risk of AD, cognitive decline, and preclinical AD, respectively^18^. The buildup of amyloid plaques and hyperphosphorylation of tau proteins, which compose neurofibrillary tangles in AD, leads to the improper functioning of neurons and their eventual death^19,20^. Therefore, the glymphatic system has the potential to exacerbate the risk of dementia and AD in T2DM patients, and its mechanisms need to be studied.

This study aims to examine the functionality of the glymphatic system in T2DM individuals by utilizing advanced imaging techniques. Magnetic resonance imaging (MRI)-based diffusion tensor imaging along the perivascular space (DTI-ALPS) index offers a non-invasive, valuable means to better understand the glymphatic system in individuals with T2DM. The DTI-ALPS index leverages the principles of DTI to quantify fluid flow dynamics along the perivascular pathways and has been used in multiple conditions^21–25^. Recent studies suggest that glymphatic dysfunction, as measured by the DTI-ALPS index, is associated with β-amyloid and tau protein deposition, neurodegeneration, and clinical progression in AD^23^. Also, literature suggest that alterations in the DTI-ALPS index are observed in several neurological and sleep-related conditions, including AD, Parkinson’s diseases, corticobasal syndrome, and obstructive sleep apnea^22,24,25^. Although these findings support the usefulness of the DTI-ALPS index to examine glymphatic function, some additional factors, such as microstructural tissue alteration may impact the DTI-ALPS index, and it should be considered as indirect measure of glymphatic system activity. The goal of our study was to examine glymphatic system function in T2DM adults in comparison to healthy controls using the DTI-ALPS index.

## Materials and methods

### Subjects

A total of 78 T2DM adults (39 male and 39 female) and 106 control subjects (53 male and 53 female) were recruited for this study. Demographic and clinical data are presented in Table 1. T2DM patients’ medication regimens were stable, and all subjects were able to lay supine for the MRI. Control subjects were healthy and had no history of hypertension or diabetes and were not on any medications known to have neurotoxic/neuroprotective effects that could potentially alter brain structures or functions (e.g. antihypertensive or psychotropic medication). T2DM patients were recruited from the UCLA Gonda Diabetes Center and healthy controls through flyer advertisements on the UCLA campus and the West Los Angeles area. Multiple conditions, including psychiatric disease (e.g., major depressive disorder, schizophrenia, and bipolar disorder), diagnosed neurological disorders (e.g., seizure history, traumatic brain injury), cardiovascular events, such as stroke or heart failure, structural chest or airway abnormalities affecting respiration, renal failure, dementia, cystic fibrosis, chronic obstructive pulmonary disease, substance dependencies, claustrophobia, body weight exceeding 160 kg (due to MRI scanner limitations), or metallic implants were considered as exclusion criteria for both T2DM and controls. For T2DM adults, exclusion criteria were confirmed through a detailed review of their electronic medical records, and for healthy controls, such details were collected through self-report and screening questionnaires. In addition, the obstructive sleep apnea (OSA) diagnosis in T2DM adults was determined from clinical chart review, and in healthy controls from screening questionnaires. All T2DM and control subjects provided written informed consent before the study, and the research protocol of this study was approved by the UCLA Institutional Review Board. In addition, all methods were performed in accordance with the relevant guidelines and regulations.

**Table 1:** Demographics and clinical variables of T2DM patients and healthy controls.

Variables	T2DM (mean ± SD) [*n* = 78]	Controls (mean ± SD) [*n* = 106]	*p* values	Effect Size (d)
Age (years)	56.5 ± 7.5	54.7 ± 6.5	0.09	0.26
Sex (Male: Female)	39:39	53:53	1.0	-
Ethnicity				-
African American	7 (9.0%)	13 (13.2%)	0.15	
Asian	16 (20.5%)	30 (28.9%)		
Hispanic	24 (30.8%)	18 (16.5%)		
White	23 (29.5%)	39 (36.4%)		
Other	7 (9.0%)	6 (5.0%)		
Unknown	1	0		
BMI (kg/m^2^)	29.45 ± 5.0	26.28 ± 4.2	< 0.001*	0.69
Heart Rate (beats/min)	77.3 ± 11.8 (*n* = 68)	71.7 ± 10.9	0.002*	0.49
Systolic BP (mmHg)	127.8 ± 15.3 (*n* = 69)	120.7 ± 17.2	0.006*	0.44
Diastolic BP (mmHg)	78.6 ± 10.4 (*n* = 69)	78.8 ± 14.2	0.92	0.02
PSQI Total PSQI Component 1 PSQI Component 2 PSQI Component 3 PSQI Component 4 PSQI Component 5 PSQI Component 6 PSQI Component 7	5.7 ± 3.6 1.01 ± 0.85 0.86 ± 0.94 0.95 ± 0.92 0.60 ± 0.93 1.23 ± 0.60 0.33 ± 0.78 0.64 ± 0.70	4.5 ± 2.8 (*n* = 105) 0.84 ± 0.68 0.82 ± 0.85 0.83 ± 0.88 0.32 ± 0.69 1.01 ± 0.43 0.29 ± 0.74 0.44 ± 0.55	0.02* 0.12 0.76 0.37 0.03* 0.006* 0.68 0.03*	0.37 0.22 0.04 0.13 0.34 0.42 0.05 0.32
ESS	6.7 ± 3.9 (*n* = 69)	5.1 ± 3.3	0.004*	0.44
Diabetes Duration (years)	10.7 ± 8.1	-		
Glycated Hemoglobin (HbA1c, mmol/L)	7.04 ± 1.3	5.3 ± 0.4 (*n* = 52)	< 0.001*	1.81
MoCA Total Visuospatial Naming Attention Language Abstraction Delayed Recall Orientation	25.9 ± 2.5 4.15 ± 0.9 2.9 ± 0.3 5.1 ± 1.1 2.2 ± 1.0 1.9 ± 0.3 3.5 ± 1.4 5.9 ± 0.2	27.1 ± 2.3 4.55 ± 0.7 3.0 ± 0.2 5.6 ± 0.8 2.5 ± 0.7 2.0 ± 0.2 3.5 ± 1.5 6.0 ± 0.1	0.001* < 0.001* 0.27 0.001* 0.009* 0.13 0.98 0.12	0.50 0.50 0.39 0.52 0.35 0.39 0 0.63

T2DM = Type 2 Diabetes Mellitus; SD = Standard Deviation; BP **=** Blood Pressure; BMI = Body Mass Index; MoCA = Montreal Cognitive Assessment; PSQI = Pittsburgh Sleep Quality Index; PSQI Component 1 = subjective sleep quality, PSQI Component 2 = sleep latency, PSQI Component 3 = sleep duration, PSQI Component 4 = habitual sleep efficiency, PSQI Component 5 = sleep disturbances, PSQI Component 6 = use of sleeping medications, PSQI Component 7 = daytime dysfunction; ESS = Epworth Sleepiness Scale; * = Statistically significant.

### Quality of sleep and daytime sleepiness

Both the T2DM and control groups completed questionnaires assessing sleep quality and daytime sleepiness levels. The Pittsburgh Sleep Quality Index (PSQI) was used to measure sleep quality, while daytime sleepiness was evaluated through the Epworth Sleepiness Scale (ESS). Both assessments are well-established tools for assessing sleep quality and daytime sleepiness (potential risk for sleep-disordered breathing). A score of 5–21 on the PSQI indicates poor sleep quality, and a score of 10 or higher on the ESS suggests excessive daytime sleepiness. Along with global PSQI scores, its seven component scores were evaluated: (1) subjective sleep quality, (2) sleep latency, (3) sleep duration, (4) sleep efficiency, (5) sleep disturbances, (6) sleep medication intake, and (7) daytime sleepiness. Scores ranged from 0 to 3 for each component, with higher scores indicating more severe sleep problems.

### Cognitive examination

Both T2DM and control subjects underwent the Montreal Cognitive Assessment (MoCA) test for rapid evaluation of multiple cognitive subdomains, including visuospatial skills, executive functions, attention, memory, language, and orientation. A global MoCA score of 26 or more was considered normal^26^. A cutoff MoCA score of 21 or less was used to exclude individuals with dementia.

### Magnetic resonance imaging

Brain imaging data were acquired using a 3.0-Tesla MRI scanner (Siemens Magnetom Prisma Fit, Erlangen, Germany), with participants positioned in a supine position. To minimize head motion, foam padding was placed on either side of the head. High-resolution T1-weighted images were acquired with a magnetization-prepared rapid acquisition gradient-echo (MPRAGE) pulse sequence, with the following parameters: repetition time (TR) = 2200 ms, echo time (TE) = 2.41 ms, inversion time = 900 ms, flip angle = 9°, matrix size = 320 × 320, field of view (FOV) = 230 × 230 mm, slice thickness = 0.9 mm, and a total of 192 slices. Proton-density (PD) and T2-weighted images were obtained in the axial plane using a dual-echo turbo spin-echo sequence (TR = 10,000 ms; TE1/TE2 = 12/124 ms; flip angle = 130°; matrix size = 256 × 256; FOV = 230 × 230 mm; slice thickness = 3.5 mm). For DTI, data were collected using single-shot echo-planar imaging with a twice-refocused spin-echo pulse sequence (TR = 12,200 ms; TE = 87 ms; flip angle = 90°; bandwidth = 1,345 Hz/pixel; matrix size = 128 × 128; FOV = 230 × 230 mm; slice thickness = 1.7 mm, b-values = 0 and 800 s/mm^2^; diffusion directions = 30).

### Visual assessment

High-resolution T1-, PD-, and T2-weighted images were visually assessed to identify any major brain abnormalities, including cysts, tumors, or significant brain infarcts. DTI images were also checked for artifacts related to imaging or head motion. No participants included in this study had major brain pathologies or imaging artifacts.

### DTI indices and ALPS measurement

**Fig. 1:**
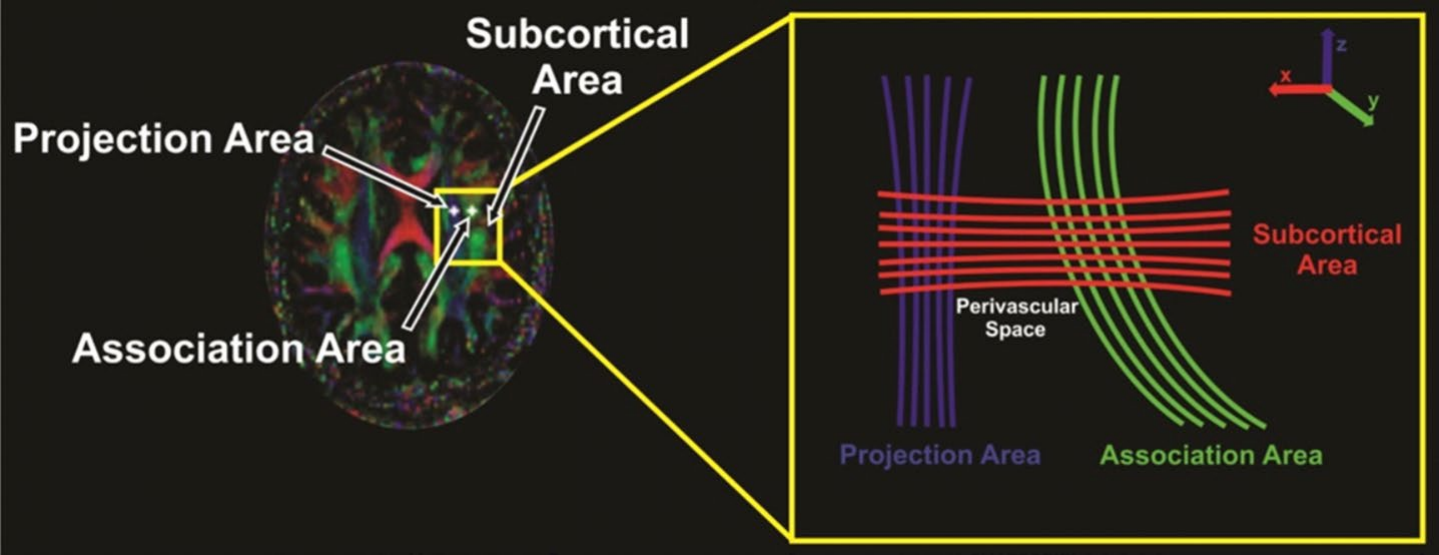
Regions of interest taken for imaging are marked with a white star and close-up panel shows different fibers running through the projection (blue), association (green), and subcortical (red) areas along with directionality.

Diffusion-weighted (b = 800 s/mm^2^) and non-diffusion-weighted (b = 0 s/mm^2^) images were used to calculate diffusion tensor matrices with the DTI-Studio software^27^. The average background noise level outside the brain parenchyma was measured from both diffusion- and non-diffusion-weighted images to facilitate the removal of non-brain regions during the tensor calculation. Diffusivity maps (D_xx_, D_yy_, D_zz_, D_xy_, D_yz_, and D_xz_) were then computed. The DTI-ALPS index was calculated following the methodology outlined in previous studies^22,28^. The index was derived by analyzing diffusivity along the direction of the perivascular space with diffusivity along projection and association fibers on an axial slice near the level of the lateral ventricles, where the medullary veins are oriented perpendicular to the ventricular wall and aligns with the x-axis, representing the perivascular space’s direction and the direction of both the projection (z-axis) and the association (y-axis) fibers are perpendicular to the direction of the perivascular space (Fig. 1).

All diffusivity maps were normalized to Montreal Neurological Institute (MNI) space. Using the unified segmentation method, non-diffusion-weighted (b0) images were normalized to MNI space, and the resulting normalization parameters were applied to all diffusivity maps. Two sets of regions of interest (ROIs) were placed in areas corresponding to the projection and association fibers at the level of the lateral ventricle body on the normalized diffusivity maps. These ROIs provided values for diffusivity parameters (D_xx_, D_yy_, D_zz_, D_xy_, D_yz_, and D_xz_) for each subject in the projection and association fibers areas, and using these values, the ALPS index was calculated as: $\:\mathrm{A}\mathrm{L}\mathrm{P}\mathrm{S}\:\mathrm{i}\mathrm{n}\mathrm{d}\mathrm{e}\mathrm{x}=\frac{({D}_{xxpro}+\:{D}_{xxasc})/2}{\left({D}_{yypro}{+D}_{zzasc}\right)/2}$ where D_xxpro_ and D_yypro_ are D_xx_ and D_yy_ in the area of projection fibers, and D_xxasc_ and D_zzasc_ are D_xx_ and D_zz_ in the association fiber areas.

### Statistical analysis

Differences in demographics and clinical variables were analyzed using the independent samples t-tests and Chi-square tests with the Statistical Package for the Social Sciences (SPSS, v 29.0, IBM Corp., Armonk, NY, United States). Diffusivity values and ALPS indices were compared between the T2DM and control groups using analysis of covariance (SPSS Software; ANCOVA; covariates, age, sex, and BMI). To further account for cognition and sleep-related variability, MoCA and ESS scores were categorized as “1” (MoCA < 26) or “0” (MoCA ≥ 26), and “1” (ESS ≥ 11) or “0” (ESS < 11), respectively. OSA status was categorized as “1” (presence) or “0” (absence). Additional ANCOVA analyses were also performed to examine differences in DTI metrics and the DTI-ALPS indices, adjusting for MoCA, ESS, and OSA status, in addition to age, sex, and BMI. The results were corrected for multiple comparisons using the Bonferroni correction. A value of *p* < 0.05 was chosen to establish statistical significance. Pearson’s correlation and partial correlation analyses (covariates: age, sex, and BMI) were performed to assess associations between sleep measures (PSQI and ESS), disease duration, HbA1c levels, and the DTI-ALPS index in T2DM adults.

## Results

### Demographic and clinical variables

Demographic and other clinical variables of T2DM and control subjects are summarized in Table 1. No significant differences in age (*p* = 0.09, d = 0.26), sex (*p* = 1.0), or ethnicity (*p* = 0.15) observed between the T2DM and control groups. However, the body mass index (*p* < 0.001, d = 0.69) was significantly higher in T2DM over controls. Resting heart rate (*p* = 0.002, d = 0.49) and systolic blood pressure (*p* = 0.006, d = 0.44) were also elevated in T2DM adults compared to healthy controls, reflecting mild cardiovascular dysregulation often associated with diabetes. The HbA1c levels (*p* < 0.001, d = 1.81) were markedly higher in T2DM over healthy controls, confirming chronic hyperglycemia in the diabetic cohort.

**Fig. 2:**
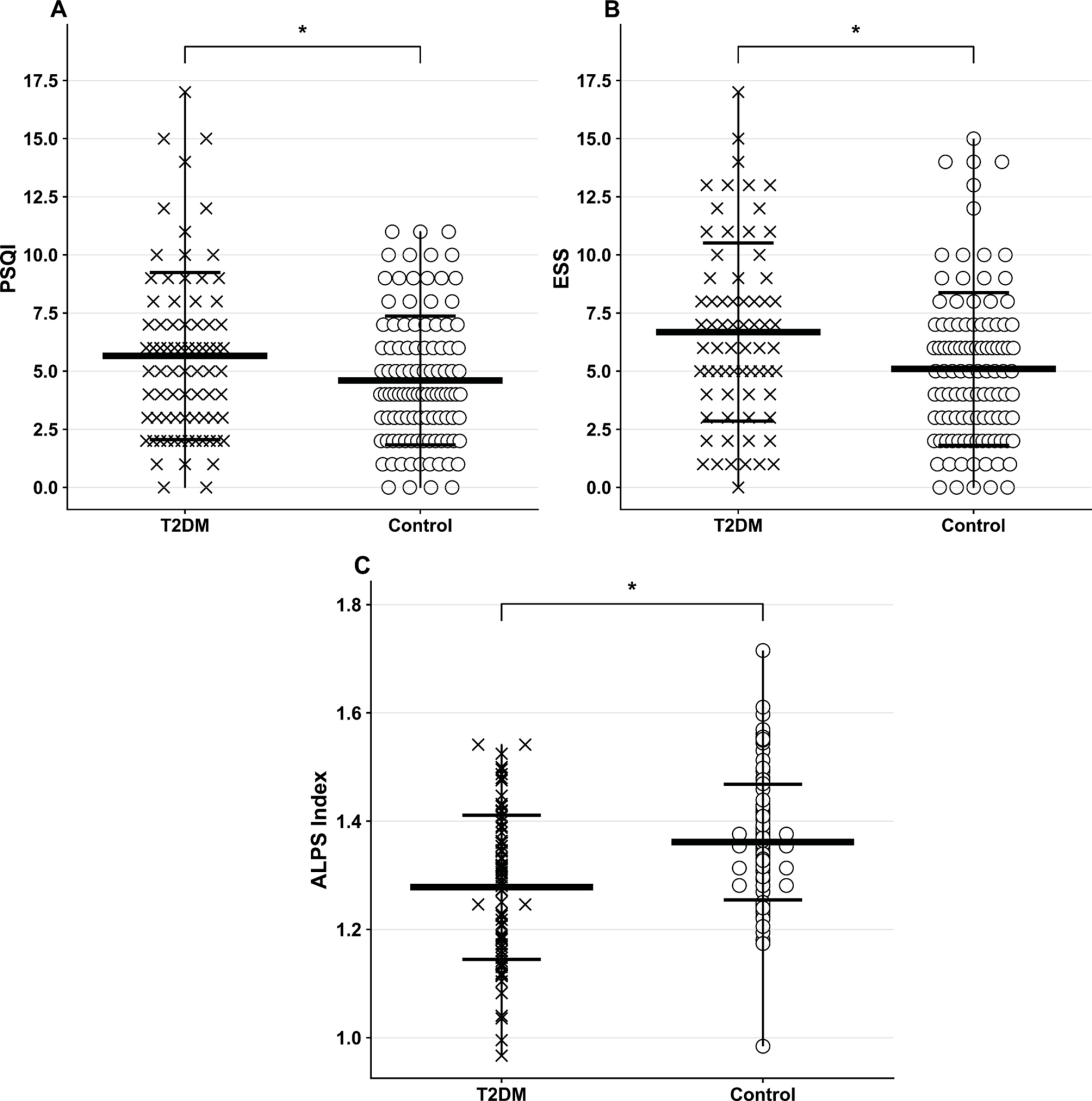
Jitter plots with overlaid mean and standard deviation bars of perivascular space indexes in T2DM patients and healthy controls: (**A**) PSQI, (**B**) ESS, and (**C**) ALPS indexes; * represents *p* < 0.05.

The ESS (*p* = 0.004, d = 0.44) and PSQI (*p* = 0.03, d = 0.37) scores were significantly increased in T2DM over control subjects. These scores are illustrated using jitter plots, which display individual ESS and PSQI scores for each participant overlaid with group means and standard deviation bars to visualize the distribution and variability within each group (Fig. 2). A higher incidence of sleep disturbance (*p* = 0.006, d = 0.42), greater daytime dysfunction (*p* = 0.03, d = 0.32), and diminished sleep efficiency (*p* = 0.03, d = 0.34) were also observed in T2DM compared to healthy controls. Global MoCA scores were significantly lower in T2DM compared to controls (*p* = 0.001, d = 0.50), with significant differences emerging in the visuospatial (*p* < 0.001, d = 0.50), attention (*p* = 0.001, d = 0.52), and language (*p* = 0.009, d = 0.35) subdomains.

Among individuals with T2DM, 41% had MoCA scores below 26, compared with 20.8% of healthy controls. Also, 16.7% of T2DM adults and 5.7% of controls had ESS scores ≥ 11. Notably, 17.9% individuals with T2DM had a documented diagnosis of OSA, while none of the controls reported OSA on their screening questionnaires. These findings highlight that T2DM adults demonstrate cognitive and sleep-related changes.

### Diffusion and ALPS indices

**Fig. 3:**
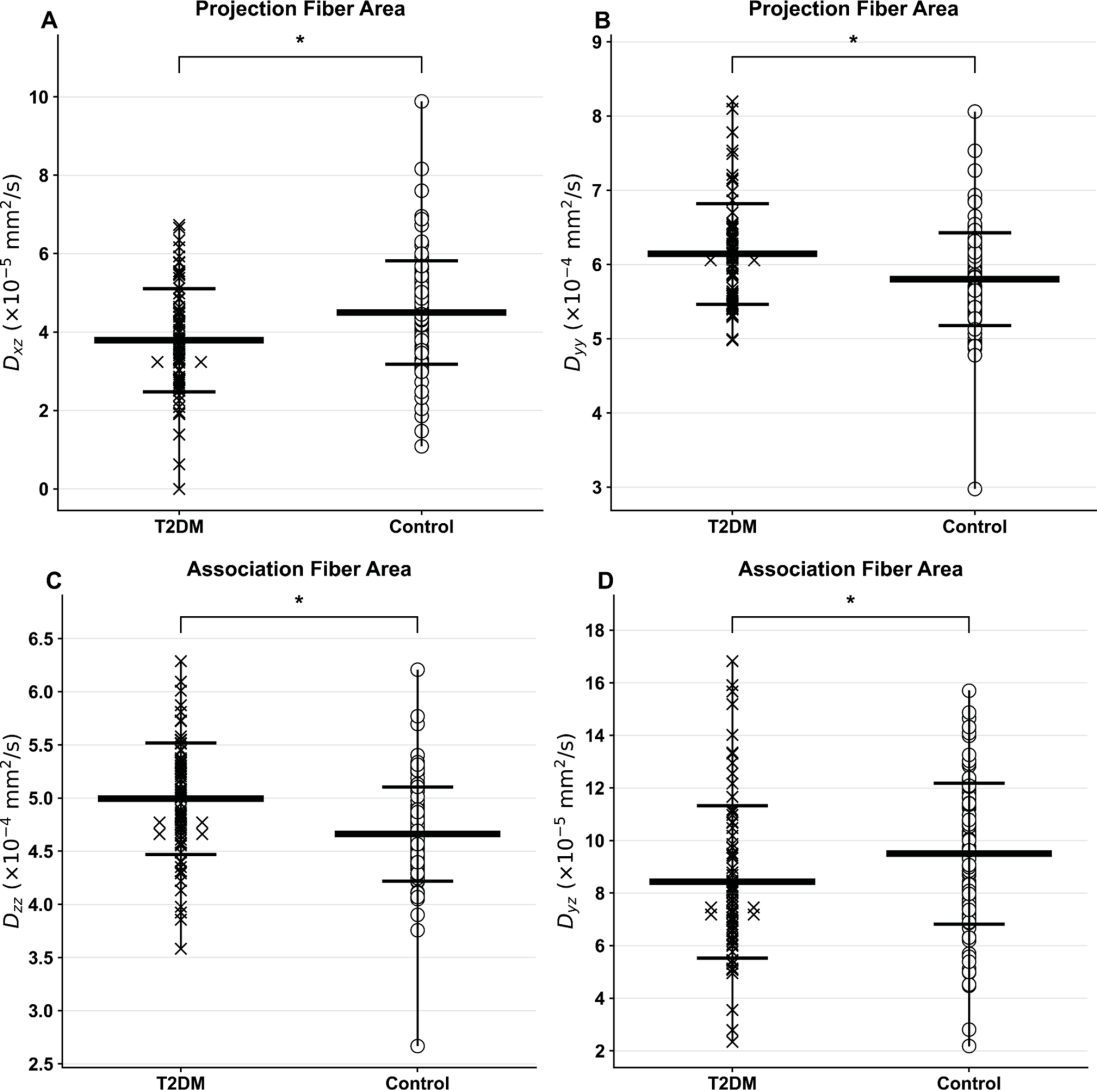
Jitter plots with mean and standard deviation bars of perivascular space indexes in T2DM patients and healthy controls: (**A**) Dxz in projection fibers, (**B**) Dyy, (C) Dzz in association fibers, and (D) Dyz; * represents *p* < 0.05.

The group-wise distributions of DTI-ALPS values are displayed as jitter plots, illustrating individual data points for all T2DM and healthy adults along with group means and standard deviation bars (Fig. 3). The DTI-ALPS index (*p* = 0.003, f = 0.25) was significantly decreased in T2DM compared to control subjects, indicating impaired glymphatic function. In addition, diffusion metrics derived from the projection and association fiber regions demonstrated significant group differences. Specifically, D_xz_ (*p* = 0.001, f = 0.5) and D_yy_ (*p* = 0.03, f = 0.17) values from the projection fiber areas, as well as D_zz_ (*p* < 0.001, f = 0.3) from the association fiber areas (Fig. 3), were significantly different between T2DM and controls (Table 2). These diffusion components are visualized using jitter plots to show inter-individual variability and group-level trends (Fig. 3). Additional ANCOVA analyses adjusted for MoCA, ESS, and OSA status, in addition to age, sex, and BMI showed significant group differences for the DTI-ALPS index and several other DTI-derived metrics (Supplementary Material, Table S1). No significant associations were found between sleep measures and the DTI-ALPS indices using Pearson’s and partial correlation analyses in T2DM individuals (Supplementary Material, Tables S2). A negative trend was observed between the DTI-ALPS indices and disease duration (*r* = −0.17, *p* = 0.14) using Pearson’s correlation in T2DM adults (Supplementary Material, Tables S3).

**Table 2:** Diffusivity and DTI-ALPS indices of T2DM patients and healthy controls.

Periventricular Projection Fiber Area (mean ± SD, x 10^− 3^ mm^2^/s)				
	T2DM	Controls	*p* values	Effect Size (f = d/2)
Dxx	0.72 ± 0.05	0.72 ± 0.05	1.0	0
Dxy	0.04 ± 0.01	0.05 ± 0.01	0.42	0.5
Dxz	0.04 ± 0.01	0.05 ± 0.01	0.001*	0.5
Dyy	0.61 ± 0.06	0.59 ± 0.06	0.03*	0.17
Dyz	0.20 ± 0.03	0.20 ± 0.03	0.26	0
Dzz	0.93 ± 0.07	0.93 ± 0.07	1.0	0

Periventricular Association Fiber Area (mean ± SD, x 10^− 3^ mm^2^/s)				
	T2DM	Controls	*p* values	Effect Size (f = d/2)
Dxx	0.69 ± 0.06	0.69 ± 0.06	0.43	0
Dxy	0.12 ± 0.03	0.12 ± 0.03	0.26	0
Dxz	0.06 ± 0.01	0.06 ± 0.01	1	0
Dyy	0.99 ± 0.06	1.01 ± 0.06	0.38	0.17
Dyz	0.08 ± 0.03	0.09 ± 0.03	0.03*	0.17
Dzz	0.50 ± 0.05	0.47 ± 0.05	< 0.001*	0.3

	T2DM	Controls	*p* values	Effect Size (f = d/2)
ALPS	1.296 ± 0.11	1.35 ± 0.11	0.003*	0.25

ALPS = analysis along the perivascular space; T2DM = Type 2 Diabetes Mellitus; SD = Standard Deviation, Dxx = diffusivity in x-direction; Dxy = diffusivity in x-y direction; Dxz = diffusivity in x-z direction; Dyy = diffusivity in y direction; Dyz = diffusivity in y-z direction; Dzz = diffusivity in z direction; * = Statistically significant.

## Discussion

We found significantly reduced DTI-ALPS indices, an indicator of impaired glymphatic system function, in adults with T2DM compared to healthy controls. In addition, the diffusivity measures along projection and association fibers were altered in individuals with T2DM. The daytime sleepiness and sleep quality, measured using ESS and PSQI, showed increased daytime sleepiness and poorer sleep quality in T2DM over healthy controls. Also, the MoCA scores in T2DM patients were lower than those of healthy controls, indicating the presence of early cognitive impairments associated with the condition. These findings suggest a critical interplay between metabolic dysregulation, glymphatic dysfunction, and sleep disturbances in T2DM adults that may contribute to cognitive decline, as observed in this study, and pose early risks for dementia and AD.

The findings of this study suggest that glymphatic system function is significantly impaired in individuals with T2DM compared to healthy controls. We used DTI-based measures to examine glymphatic system status. Although the ALPS index is not a direct measure of glymphatic system activity, its validity has been supported in other disease conditions through comparison with other imaging-based methods assessing glymphatic function^29^. However, alternative mechanisms, including microstructural brain tissue changes, should also be considered when interpreting reduced DTI-ALPS values between T2DM and controls groups. The glymphatic system plays a key role in clearing metabolic waste and maintaining brain tissue function, and impaired glymphatic clearance may contribute to the increased vulnerability of the brain in T2DM adults, including tissue changes^3–5^, which may exacerbate neurodegenerative and cognitive disorders. Since metabolic dysregulation, inflammation, and vascular dysfunction are prevalent in T2DM adults^30^, the compromised glymphatic function observed in our study may be an underlying factor linking the condition with increased neurocognitive risks.

Additionally, this study revealed that individuals with T2DM had higher ESS and worse PSQI scores, indicating greater daytime sleepiness and poorer sleep quality compared to healthy controls. Sleep is crucial for optimal glymphatic system function, as it is more active during sleep and facilitates cerebrospinal fluid flow, as well as enhances waste clearance from the brain parenchyma^10,31^. The poor sleep quality observed in T2DM patients may further exacerbate glymphatic dysfunction, and this interplay creates a compounding cycle in which impaired sleep leads to reduced waste clearance, which in turn may contribute to cognitive decline^32^. These findings suggest that sleep disruptions in T2DM could be a driving factor of glymphatic system impairment and associated cognitive risks. Understanding this interrelationship highlights the importance of the glymphatic system in T2DM adults and its potential as a therapeutic target to mitigate early risks of AD and other neurodegenerative conditions in this high-risk patient population^32–34^.

Although T2DM individuals demonstrated significantly lower DTI-ALPS values over healthy controls in the fully-adjusted statistical model, including adjustment for MoCA, there were no significant correlations between DTI-ALPS and PSQI, ESS, or HbA1c within T2DM adults. These findings suggest that the observed group differences in DTI-ALPS indices may not be directly driven by severity of sleep disturbances or glycemic status alone. Since lower MoCA scores were observed in T2DM adults, an alternative explanation may include early microstructural brain tissue changes associated with subtle cognitive decline and contribute to the reduced ALPS indices. Alterations in DTI metrics, such as fractional anisotropy and mean diffusivity, are well documented in mild cognitive impairment (MCI) and early neurodegeneration^35–37^, and similar processes may influence the DTI-ALPS indices. Thus, while impaired glymphatic function remains a strong interpretation, the potential contribution of early neurodegenerative or microstructural brain changes should also be considered. Longitudinal studies incorporating multimodal imaging and comprehensive cognitive assessments will be essential to disentangle such mechanisms.

Recent animal studies investigating glymphatic system function in T2DM models have demonstrated alterations in the MRI markers of brain glymphatic measurements at both an early and advanced stage of diabetes, suggesting a sensitive marker that could serve as an early diagnostic indicator for T2DM-associated neurovascular damage and cognitive decline^15,38^. In addition, the dependency of glymphatic clearance and cerebrospinal fluid-interstitial fluid exchange on the aquaporin-4 water channel in different neurological conditions is well known^13,39–42^. The animal model of T2DM showed a decreasing trend in aquaporin-4 expression with increased disease severity of the condition, which indicates a similar mechanism may underlie glymphatic dysfunction in T2DM patients and suggests that consideration of the aquaporin-4 water channel is crucial for assessment of the glymphatic system status.

Previous studies in T2DM and metabolic syndrome have shown an association with increased neuroinflammation, oxidative stress, and vascular abnormalities^43–45^, all of these can hinder glymphatic system functionality^46–48^. Such disruptions may interfere with the clearance of β-amyloid and tau protein, leading to their accumulation and an increased risk of neurodegeneration. Our study supports the evidence that metabolic and vascular dysfunctions in T2DM are likely contributors to reduced glymphatic system efficiency. In our study, the glymphatic impairment and high ESS and PSQI scores suggest that metabolic disturbances in T2DM may not only affect peripheral organs, but also disrupt key processes within the brain, further reinforcing the need for targeted interventions to address glymphatic function in adults with T2DM.

Moreover, this study emphasizes the potential role of improving sleep in mitigating glymphatic dysfunction in T2DM patients. Given the established connection between glymphatic activity and sleep^10,22^, therapeutic approaches that improve sleep quality, such as cognitive-behavioral therapy, lifestyle changes, and possibly medications targeting sleep architecture, may support glymphatic function. These interventions may be particularly beneficial for individuals with T2DM who exhibit poor sleep quality and high daytime sleepiness, as improving sleep may enhance waste clearance and reduce the neurocognitive burden associated with individuals with T2DM.

### Limitations

This study has several limitations that should be acknowledged. The DTI-ALPS index represents an indirect measure of glymphatic function and therefore needs to be validated by pathophysiological studies. Although the ALPS index and intrathecal contrast administration methods have shown strong correlations for evaluating glymphatic system function in cerebral small vessel disease^29^, future studies of MRI with intrathecal contrast agents could help validate and extend these findings in T2DM. T2DM is associated with increased cerebrovascular disease, including white matter hyperintensities that are known to influence DTI measures. However, our study did not acquire FLAIR images, which precluded the quantification of white matter hyperintensities and their inclusion in the statistical models. Although our sample size was relatively large compared to prior DTI-ALPS studies, studies with larger sample sizes are needed to further elucidate the relationships between sleep measures, HbA1c levels, and the DTI-ALPS indices. Another limitation of this study is the use of a relatively low b-value (800 s/mm²) for DTI acquisition. This was a deliberate trade-off to optimize the signal-to-noise ratio (SNR) and minimize motion-related artifacts, which can significantly affect data quality in clinical populations, as well as to avoid biexponential signal decay issues. Although higher b-values may enhance sensitivity to restricted diffusion, they often result in reduced SNR and unstable tensor fitting. A moderate b-value in the range of 700–900 s/mm² has been widely adopted in prior DTI^4,49,50^ and DTI-ALPS^22^ studies for reliable estimation of diffusion metrics. However, the optimal b-value for evaluating the DTI-ALPS index remains to be established. Also, sleep quality was assessed using the PSQI, which primarily reflects subjective sleep perception and may not strongly correlate with objective measures, including actigraphy or polysomnography. Polysomnographic data were not available for all participants, which may limit the precision of sleep-related inferences. However, additional analyses were performed adjusting for the presence of OSA, as identified from clinical records, and the results are presented in the Supplementary Material (Table S1).

## Conclusions

This study demonstrates significantly reduced DTI-ALPS index values in individuals with T2DM compared to healthy controls, suggesting impaired glymphatic system function. Higher daytime sleepiness and worse sleep quality in T2DM patients further highlight the multifaceted impact of metabolic dysregulation on the glymphatic system. The abnormal MoCA scores observed in T2DM patients emphasize the cognitive impairments associated with glymphatic dysfunction and sleep disturbances. The observed glymphatic dysfunction, potentially driven by metabolic, vascular, and inflammatory abnormalities, suggests a mechanism linking T2DM with cognitive decline and highlights the importance of targeting the glymphatic system as a therapeutic strategy. Sleep-targeted therapies, such as cognitive-behavioral therapy, lifestyle modifications, and sleep-promoting medications, hold promise for mitigating glymphatic dysfunction and enhancing waste clearance in this high-risk population. The findings pave the way for research into therapeutic strategies that could improve glymphatic function and protect against neurodegenerative disorders and early risk of dementia and AD in adults with T2DM.

## Supplementary Information

Below is the link to the electronic supplementary material.

Supplementary Material 1

## Data Availability

The datasets generated during and/or analyzed in the current study are available from the corresponding author upon reasonable request.
