# Supplementary material for: Glymphatic system impairment in type II diabetes mellitus adults
Source: Sci Rep. 2026 Feb 4;16:7286. doi: 10.1038/s41598-026-36573-4 (PMC12923780; doi:10.1038/s41598-026-36573-4)
Supplement: Supplementary file 1 — Supplementary Material 1 [file 41598_2026_36573_MOESM1_ESM.docx]

**Table S1:** Significant values from ANCOVA analyses between T2DM (n = 78) and healthy controls (n = 106) for DTI metrics (periventricular projection and association fiber area) and the DTI-ALPS index with age, sex, BMI, MoCA, ESS (n = 69, T2DM), and OSA status as covariates.

| **Periventricular Projection Fiber Area** | | | | | | | | | |
| --- | --- | --- | --- | --- | --- | --- | --- | --- | --- |
|  | **p values** (covariates: Age, sex, BMI) | | **p values** (covariates: Age, sex, BMI, MoCA) | | **p values**  (covariates: Age, sex, BMI, ESS) | | **p values**  (covariates: Age, sex, BMI, OSA) | **p values**  (covariates: Age, sex, BMI, MoCA, ESS, OSA) | |
| **Dxx** | 1.0 | | 1.0 | | 1.0 | | 1.0 | 1.0 | |
| **Dxy** | 0.42 | | 0.38 | | 1.0 | | 0.22 | 0.55 | |
| **Dxz** | 0.001* | | 0.001* | | 0.003* | | <0.001* | 0.002* | |
| **Dyy** | 0.03* | | 0.035* | | 0.085 | | 0.06 | 0.16 | |
| **Dyz** | 0.26 | | 0.18 | | 0.48 | | 0.16 | 0.23 | |
| **Dzz** | 1.0 | | 0.89 | | 0.81 | | 0.66 | 0.42 | |
| **Periventricular Association Fiber Area** | | | | | | | | | |
|  | **p values** (covariates: Age, sex, BMI) | | **p values** (covariates: Age, sex, BMI, MoCA) | | **p values**  (covariates: Age, sex, BMI, ESS) | | **p values**  (covariates: Age, sex, BMI, OSA) | **p values**  (covariates: Age, sex, BMI, MoCA, ESS, OSA) | |
| **Dxx** | 0.43 | | 0.37 | | 0.58 | | 0.27 | 0.35 | |
| **Dxy** | 0.26 | | 0.16 | | 0.35 | | 0.17 | 0.17 | |
| **Dxz** | 1.00 | | 1.00 | | 0.75 | | 1.00 | 1.00 | |
| **Dyy** | 0.38 | | 0.47 | | 0.63 | | 0.47 | 0.89 | |
| **Dyz** | 0.03* | | 0.02* | | 0.16 | | 0.08 | 0.29 | |
| **Dzz** | <0.001* | | <0.001* | | 0.002* | | <0.001* | <0.001* | |
| **ALPS Index** | | | | | | | | | |
| **p values** (covariates: Age, sex, BMI) | | **p values** (covariates: Age, sex, BMI, MoCA) | | **p values**  (covariates: Age, sex, BMI, ESS) | | **p values**  (covariates: Age, sex, BMI, OSA) | | | **p values**  (covariates: Age, sex, BMI, MoCA, ESS, OSA) |
| 0.003* | | 0.003* | | 0.009* | | 0.005* | | | 0.017* |

**Table legends:** ANCOVA = Analysis of covariance; DTI = Diffusion tensor imaging; T2DM = Type 2 diabetes mellitus; ALPS = Analysis along the perivascular space; BMI = Body mass index; MoCA = Montreal cognitive assessment; ESS = Epworth sleepiness scale; OSA = Obstructive sleep apnea; * = Statistically significant.

**Table S2:** Correlations between sleep measures (PSQI and ESS) and the DTI-ALPS indices.

| **Variables** | **Correlation Type** | **r (p values)** |
| --- | --- | --- |
| PSQI vs DTI-ALPS | Pearson correlation  (n=78, T2DM) | -0.13 (0.26) |
| PSQI vs DTI-ALPS | Partial correlation  (n=78, T2DM; covariates: age, BMI, and sex) | -0.13 (0.27) |
| ESS vs DTI-ALPS: | Pearson correlation  (n=69, T2DM) | -0.003 (0.98) |
| ESS vs DTI-ALPS: | Partial correlation  (n=69, T2DM; covariates: age, BMI, and sex) | 0.006 (0.96) |

**Table legends:** PSQI = Pittsburgh sleep quality index; ESS = Epworth sleepiness scale; ALPS = Analysis along the perivascular space; T2DM = Type 2 diabetes mellitus; BMI = Body mass index; DTI= Diffusion tensor imaging.

**Table S3:** Correlations between DTI-ALPS indices, disease durations, and HbA1c levels in T2DM adults.

| **Variables** | **Correlation Type** | **r (p values)** |
| --- | --- | --- |
| DTI-ALPS vs Diabetes duration | Pearson correlation  (n=78, T2DM) | -0.17 (0.14) |
| DTI-ALPS vs Diabetes duration | Partial correlation  (n=78, T2DM; covariates: age, BMI, and sex) | -0.09 (0.43) |
| DTI-ALPS vs HbA1c | Pearson correlation  (n=78, T2DM) | 0.04 (0.73) |
| DTI-ALPS vs HbA1c | Partial correlation  (n=78, T2DM; covariates: age, BMI, and sex) | 0.15 (0.21) |

**Table legends:** DTI = Diffusion tensor imaging; ALPS = Analysis along the perivascular space; T2DM = Type 2 diabetes mellitus.
